# Evaluation of a 3D-printed nanohybrid resin composite versus a milled resin composite for flexural strength, wear and color stability

**DOI:** 10.1186/s12903-025-05861-2

**Published:** 2025-04-15

**Authors:** Ghydaa A. Mahran, Ahmed El-Banna, Dalia I. El-Korashy

**Affiliations:** 1 Department of Biomaterials, Faculty of Dentistry, Suez University, P.O. Box: 43221, Suez, Egypt; 2 Department of Biomaterials, Faculty of Dentistry, Ain Shams University, Cairo, Egypt

**Keywords:** CAD/CAM, 3D-printing, Milling, Thermocycling, Wear, Color stability

## Abstract

**Background:**

Controversial properties and performance of commercially available 3D-printed resin composite for permanent restorations. So, the purpose of this study was to assess the flexural strength, microhardness, wear, and color stability of 3D-printed versus milled nanohybrid resin composites for permanent restoration.

**Methods:**

A total of 70 samples of nanohybrid resin composites were used; 38 bar-shaped (14 mm ⋅ 2 mm ⋅ 2 mm) and 32 disc-shaped samples (10 mm ⋅ 2 mm) of Tetric CAD^™^ blocks (TC) and Flexcera Smile Ultra plus^™^ (FSU) were fabricated (*n* = 35). Flexural properties were tested using 3-point bending test. The Vickers test was used for microhardness evaluation. Volumetric wear analysis and color changes were assessed after simulated aging via Geomagic Control X software and a Vita Easyshade spectrophotometer, respectively. Color changes were calculated via the CIEDE2000 formula. A paired t-test was used for dependent variable analysis, and the Mann‒Whitney U test was used for independent variables (α = 0.05).

**Results:**

TC resulted in significantly higher flexural strength (247.7 ± 29.1 MPa) and microhardness (94.6 ± 3 gf/um^2^) than did FSU (97.2 ± 10.2 MPa and 31 ± 4.6 gf/um^2,^ respectively) (*P* < 0.0001). Compared with FSU (–36.3 mm^3^), TC resulted in significantly lower wear rates (–17.6 mm^3^)(*P* < 0.0001). TC had a ΔE00 value of 2.4 ± 0.5, whereas FSU had a value of 2.1 ± 0.7 (*P* = 0.532), with no significant difference between the groups, but both values were above the acceptability limit (1.8).

**Conclusions:**

Compared with 3D-printed nanohybrid resin composites, milled nanohybrid resin composites have better flexural strength, microhardness and wear properties.

**Clinical relevance:**

Milled nanohybrid resin composites exhibit superior flexural strength, microhardness, and wear resistance, making them potentially more durable for clinical dental restorations compared to 3D-printed nanohybrid resin composites.

**Supplementary Information:**

The online version contains supplementary material available at 10.1186/s12903-025-05861-2.

## Background

Extensive hard tissue loss caused by caries, root canal treatment, wear or fractures requires indirect restorative procedures to restore the function and esthetic quality of the teeth [1]. Ongoing advancements in technology have led to the widespread use of CAD/CAM systems in laboratories and clinics for the fabrication of indirect high-quality restorations with reduced time and effort [1, 2]. The primary production technique is the subtractive manufacturing of solid materials such as blocks and discs. Polymer-infiltrated ceramic networks and resin nanoceramic materials have been developed as alternatives to glass ceramics, aiming to combine the favorable properties of ceramics and resin composites [2, 3]. These materials offer several advantages, including mechanical properties similar to those of natural teeth, ease of fabrication, and potential for intraoral modification or repair [4]. However, the subtractive method has limitations, such as the restricted motion range of the cutting device and the size of the cutting bur, which limits the millable shapes [5, 6]. This process results in material waste, as the remaining portion of the block is often unused, and recycling the excess material is challenging [7]. Additionally, the milling process can cause significant wear of the cutting tools and may induce microscopic cracks that weaken the restoration over time. Moreover, marginal defects can occur due to material chipping in sharp areas of the preparation [8].

Advancements in additive manufacturing have led to the construction of 3D-objects by sequentially depositing them layer by layer, thereby enabling the creation of complex structures [9, 10]. Compared with conventional milling methods, 3D-printing preserves more material; consequently, it could be considered a promising tool to overcome some of the limitations of the milling process [11]. Although the accuracy of 3D-printing can vary significantly between and within laboratories, it generally falls within the accuracy range of conventional CAD/CAM manufacturing methods [12]. Among the various additive manufacturing techniques, digital light processing (DLP) and stereolithography (SLA) are the most promising for producing accurate designs with fine surface finish [13, 14]. The current generation of 3D-printers is lighter, cheaper and smaller, offering advantages such as the ability to create larger and more complex objects, avoid material waste and simultaneously produce multiple items, making them more accessible to chairside digital dentistry than ever before [11]. In both industrial and chairside settings, 3D-printers can work with various materials, including polymers, ceramics, and metals [13, 15]. However, these methods have several disadvantages, including high costs and time-consuming postprocessing procedures [4]. It has been reported that the development of new materials and technologies will be the future trend of 3D-printing in dentistry [11].

Recently, resin composites incorporating ceramic particles have emerged as a viable option for 3D-printed, single-tooth definitive restorations [2, 13, 16]. Although the mechanical and physical properties of these materials have been investigated, their performance remains a subject of debate [13–15, 17–26]. The potential benefits of 3D-printed materials for permanent restorations have drawn considerable attention from dental researchers, suggesting their possible use as an alternative to subtractive manufacturing techniques [17, 27–34]. However, further independent studies are necessary to assess the long-term behavior of these materials before they can be broadly recommended for all types of single-tooth permanent restorations.

Accordingly, it was of interest to assess the mechanical and physical properties of commercially available 3D-printed resin composite materials indicated for single-unit permanent restorations before and after aging. The null hypothesis tested was that there would be no difference in flexural strength, microhardness, wear resistance, or color stability between 3D-printed and milled nanohybrid resin composite materials, as indicated for single-unit permanent restorations.

## Methods

### Materials

**Fig. 1:**
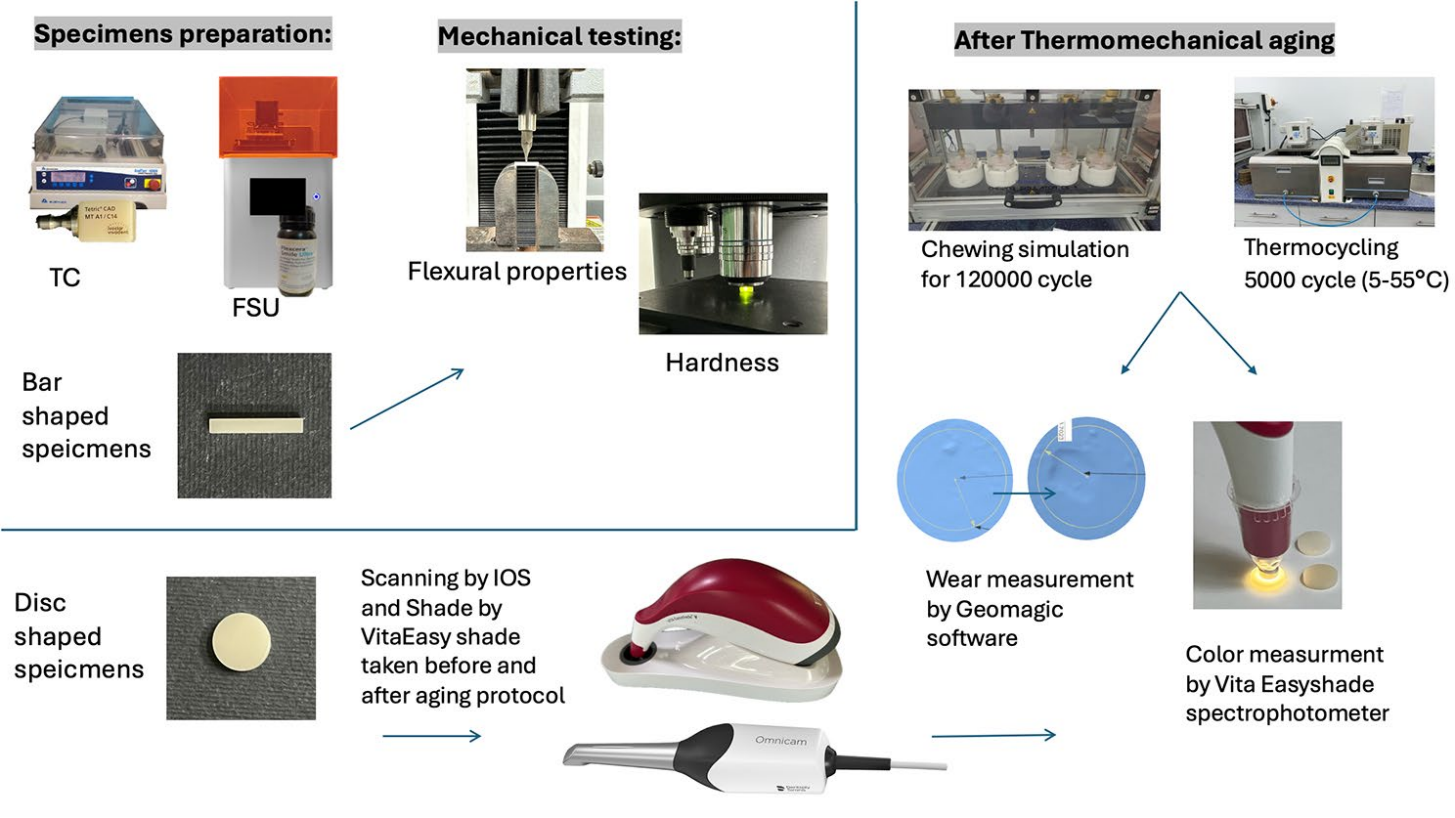
Graphical abstract showing the design of this study

The materials used in the study, compositions, lot numbers, forms of supply and manufacturers are presented in (Table 1), with a graphical abstract showing the design for this study in (Fig. 1).

**Table 1:** Materials used in this study, compositions, lot numbers, supply forms and manufacturers

Material Brand Name/Shade	Composition*	Lot No.	Form of supply	Manufacturer
CAD/CAM Nanohybrid Resin Composite block (Tetric CAD™) (TC) Shade A1	Barium glass filler 64%, silicon dioxide 7.1%, dimethacrylates (bis-GMA, bis-EMA, UDMA, TEGDMA) 28.4%, additives and pigments 0.5%.	Z00N05	Block (size = C14).	Ivoclar vivadent, Schaan, Germany.
3D-Printed Nanohybrid Resin Composite Material (Flexcera Smile Ultra Plus™) (FSU) Shade A1	Diphenyl(2,4,6-trimethylbenzoyl) phosphine oxide, methacrylate monomer, methacrylic oligomer & inorganic fillers.	310122b	Liquid resin bottle.	Desktop Health Newport Beach, California, USA.

*Compositions given for the TC were taken from the literature, whereas the FSU were taken from the manufacturer data sheet and no available data from the literature about the FSU

UMDA: urethane dimethacrylate; TEGDMA: triethylene glycol dimethacrylate; bis-GMA: bisphenol A diglycidylether methacrylate; bis-EMA: ethoxylate bisphenol-A dimethacrylate

### Sample size calculation

The sample size was determined by t-test using a statistical power analysis software program (G* Power 3.1.9.3; Heinrich Heine University Dusseldorf). Accordingly, *n* = 35 samples per group provided a power of 0.8 at α = 0.05 [1].

### Samples preparation and grouping

Two nanohybrid resin composite materials were used in this study; milled Tetric CAD^™^ blocks (Group 1:TC) and 3D-printed Flexcera Smile Ultra Plus^™^ (Group 2:FSU). TC^™^ (Ivoclar Vivadent; Schaan, Germany) is delivered as blocks for milling, whereas Flexcera Smile Ultra plus^™^ (Desktop Health; California, USA) is a liquid resin material used for 3D-printing. The compositions of the tested materials are shown in (Table 1). Thirty-eight bars-shaped (14 mm ⋅ 2 mm ⋅ 2 mm) and 32 disc-shaped (10 mm ⋅ 2 mm) samples were prepared. For the preparation of Group 1 samples, TC blocks were cut using a low-speed water-cooled diamond saw (linear precision saw; Isomet 4000, Buehler, Germany) to obtain the intended specimen shapes (bar/disc). For the disc-shaped samples, the block was first milled to a 10 mm diameter cylinder with a lathe cut machine [28]. For Group 2, 3D-printed FSU samples were virtually designed using the Chitubox 1.9.0 software and saved as standard tessellation language (STL) files. FSU samples were printed using an Envision One cDLM HT 3D-printer (Envisiontec GmbH, Gladbeck, Germany) at a 0-degree orientation and 50 μm layer thickness according to the respective manufacturer’s recommendations [35]. After printing process, the samples were subjected to the postcuring process in Otoflash G171 (NK-Optik, Baierbunn, Germany) with 2⋅3000 flashes on each side to ensure polymerization and monomer conversion. The samples in the 3D-printed group were cleaned with isopropyl alcohol (99%) for 5 min, and all the supporting structures were removed with a scalpel [35]. The samples in both groups were finished with wet silicon carbide (400 ISO/FEPA, average grain size 35 μm) [4]. The final thickness of each sample was verified (with an accuracy of ± 0.01) through a digital caliper (Mitutoyo Digimatic; IP65 micrometer, Kawasaki, Japan), then samples were cleaned in distilled water. The samples were tested 24 h after their preparation. Bar-shaped samples (*n* = 32) were used to measure the flexural properties and microhardness, whereas disc-shaped samples (*n* = 32) were used for wear and color change measurements after simulated aging protocol.

### Microstructure characterization

Three randomly selected bar samples of each tested material (*n* = 3) were used for microstructure and chemical compositional analysis. The samples were mounted on aluminum stubs and examined via an environmental scanning electron microscope (ESEM; FEI Quanta 3D-200i FEG) at magnifications of 1000, and 4000X with an accelerating voltage of 20–30 kV in back-scattered electron mode under low vacuum. Energy dispersive X-ray spectroscopy (EDX-Thermo Fisher pathfinder) operated under conditions of low vacuum with a working distance of 15–17 mm was performed on different regions per cross section. ESEM images were obtained to qualitatively investigate the filler distribution and morphology, whereas EDX is a semiquantitative assessment of the chemical composition.

### Flexural strength and modulus tests

The flexural strength and modulus of the bar-shaped samples (*n* = 8) were assessed by three-point bending tests in a universal testing machine (Instron model 3345, Norwood, MA, USA**)** with a support span of 12 mm and a crosshead speed of 1 mm/min at a 5 kN load cell. The maximum loads were obtained, and the flexural strength (σ) was calculated by computer software (Bluehill, Norwood, MA, USA) [10, 18, 36–41].

### Surface microhardness test

The microhardness of the bar-shaped samples (*n* = 8 for each group) was measured using a Vickers indenter tester (Wilson hardness tester model; TUKON 1102, Germany) with a load of 980.7 mN (HV 0.1) and a dwell time of 10 s was used. Three indentations were applied at random locations for each sample. After the load is removed, the indentation is focused on the magnifying eye piece, and the two impressions are measured, usually to the nearest 0.1 μm with a micrometer, and averaged. The software automatically calculated the hardness value (HV) as HV 01 in gf/ µm^2^ by the following equation: $\:\text{H}\text{V}=1854.4\:\text{L}/\text{d}$^2^, where L represents the load in gf and d represents the average diagonal in µm^2^ [42].

### Aging protocol

Thermocycling and chewing simulations were performed for the disc-shaped samples through a Thermocycler 1100 (SD; Mechatronik) with distilled water at 5–55 °C and a dwell time of 30 s for a total of 5000 cycles [14, 22, 27, 43]. The specimens were then subjected to a dual-axis chewing simulator (SD Mechatronik; CS-4), with a loading parameter of 50 N, 0.5-mm indentation, and vertical and horizontal strokes for 120,000 cycles at 1.4 Hz, simulating 6 months of clinical service [13, 31, 43, 44].

### Volumetric wear analysis

Geomagic Control X software 2023.3.0 (3D-systems; Rock Hill, SC, USA) was used for wear analysis of the disc-shaped samples (*n* = 8), which were digitally scanned using an intraoral scanner (Omnicam; Dentsply Sirona, Germany). Scans were made before and after the samples were subjected to the aging protocol. The scans were imported as STL files into Geomagic Control X software for measuring volumetric wear loss by subtracting specimen volumes after wear testing from pretest values, as mentioned in other studies [31, 32, 43, 45, 46].

### Color change measurement

Color parameters (L*, a*, b*, c, h) were measured for the disc samples (*n* = 8) via a digital spectrophotometer (VITA Easyshade; Vita Zahnfabrik, Dentsply, Germany) [19, 20, 23, 27, 47–49]. The spectrophotometer was recalibrated before each measurement, and all measurements were performed by a single investigator under the same brightness conditions. Shade was assessed before and after aging. The color change was detected via CIEDE2000 according to the following formula:

$$\Delta {\text{E}}00 = \sqrt {\begin{array}{*{20}{c}}{\left( {\Delta {\text{L}}/{\text{KLSL}}} \right)2 + \left( {\Delta {\text{C}}/{\text{KCSC}}} \right)2 + \left( {\Delta {\text{H}}/{\text{KHSH}}} \right)2} \\{ + {\text{RT}}\left( {\Delta {\text{C}}/{\text{KCSC}}} \right)\left( {\Delta {\text{H}}/{\text{KHSH}}} \right)}\end{array}} $$

where ∆L, ∆C, and ∆H are the distinctions in terms of lightness, chroma and hue, respectively, for each sample before and after aging [4]. Color differences were assessed based on previously published data via the 50:50% color perceptibility (PT00 = 0.81) and acceptability (AT00 = 1.81) thresholds [50].

### Statistical analysis

The data were confirmed for a normal distribution via the Kolmogorov‒Smirnov test. The data were subsequently analyzed with SPSS 20^®^ (IBM SPSS Statistics, V25.0; IBM), and the results are presented as the mean and standard deviation (SD). A paired t-test was performed to evaluate the level of significance between dependent variables, whereas an independent t-test (Mann‒Whitney U test) was performed to evaluate the level of significance between different independent variables.

## Results

### Microstructure characterization

**Fig. 2:**
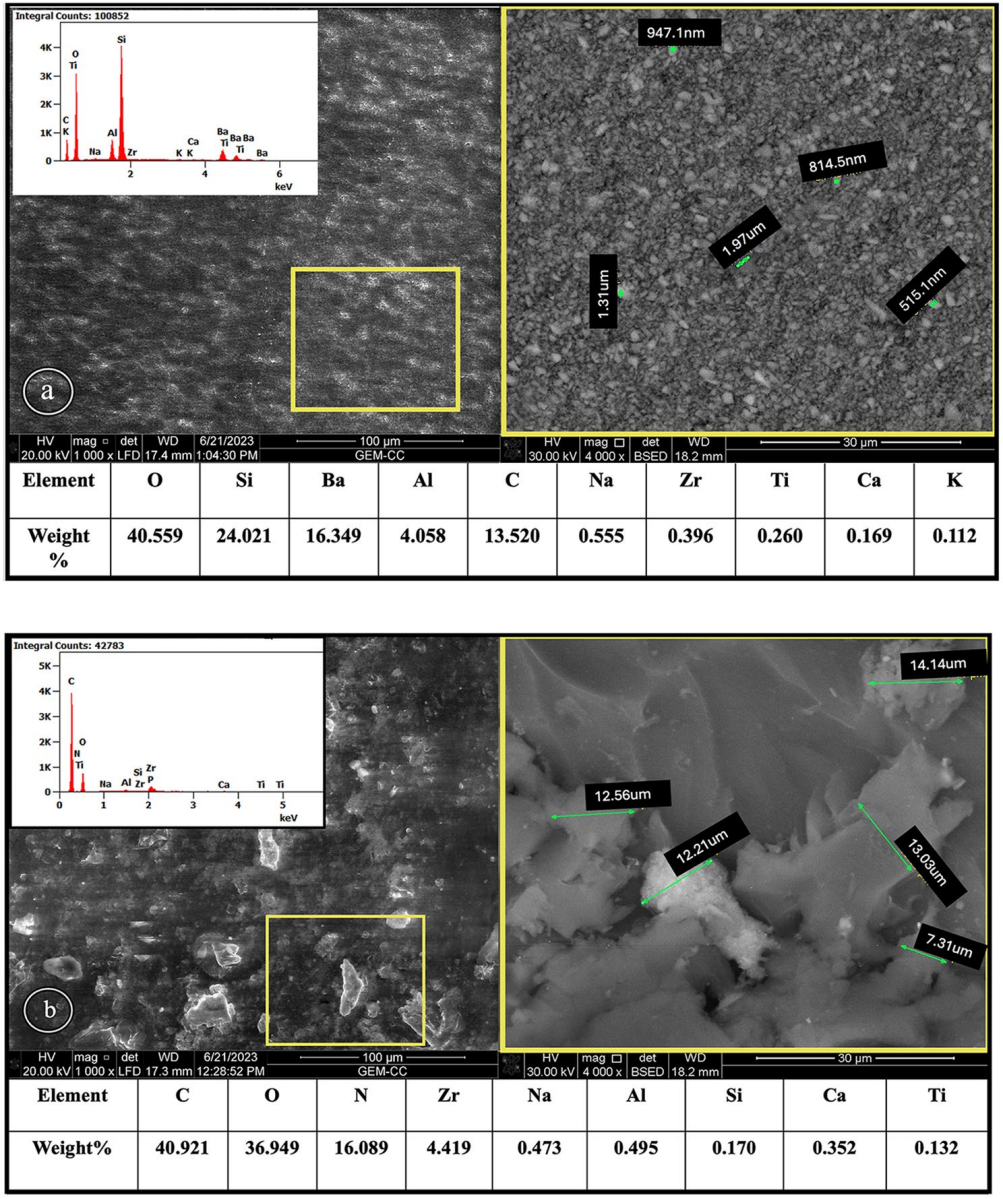
SEM-EDX results for TC (**a**) and FSU (**b**) at 1000× mag, 4000× mag, LFD and BSED modes, showing the filler size distribution and morphology, while the EDX graph and representative table show the chemical composition (wt%). **a**, Tetric CAD^™^. **b**, Flexcera Smile Ultra plus ^™^

TC was characterized by a homogeneous distribution of numerous inorganic fillers of varying sizes and shapes (~ 500 nm to 2 μm) embedded within a connecting organic matrix. In contrast, FSU resulted in larger, irregularly shaped fillers (~ 7–14 μm) that were heterogeneously and unevenly dispersed within an organic matrix. EDX analysis of TC revealed that approximately 95% of its chemical composition consisted of O, Si, Ba, and C, whereas FSU was primarily composed of C, O, and N, constituting approximately 94% of its composition, as depicted in (Figs. 2a, b, and in appendix).

### Flexural strength, modulus and surface microhardness

The flexural strength and modulus were significantly greater for the TC group (247.7 ± 29.1 MPa and 7.5 ± 0.8 GPa, respectively) than for the FSU group (97.2 ± 10.2 MPa and 1.7 ± 0.3 GPa, respectively) (*P =* 0.00214, *P* < 0.0001 respectively). Moreover, the TC group presented higher surface microhardness values (94.6 ± 3 gf/ µm^2^) than the FSU group (31 ± 4.6 gf/µm^2^) (*P* < 0.0001), as presented in (Table 2).

**Table 2:** Mean ± standard deviation values of flexural strength (MPa), flexural modulus (GPa) and microhardness (gf/µm2) for milled (TC) and 3D-printed (FSU) nanohybrid resin composites

	Flexural strength (MPa)	Flexural modulus (GPa)	Microhardness (gf/um^2^)
TC	247.7 ± 29.1	7.5 ± 0.8	94.6 ± 3
FSU	97.2 ± 10.2	1.7 ± 0.3	31 ± 4.6
*P-*value	0.00214*	< 0.0001*	< 0.0001*

*Indicates a significant difference according to the Mann‒Whitney U test for the flexural strength test results and the independent t-test for the flexural modulus and microhardness test results

### Volumetric wear analysis

**Fig. 3:**
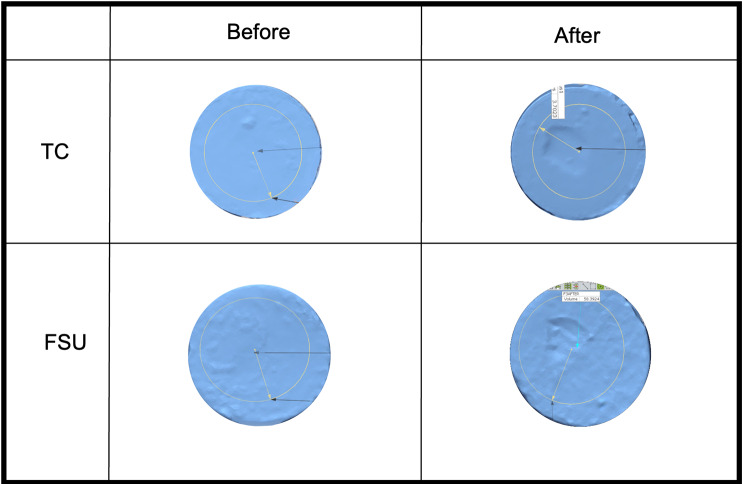
Images from Geomagic software showing samples from the TC and FSU groups before and after thermomechanical aging

The volumetric wear results revealed that both materials were subjected to wear after thermomechanical aging. However, the amount of volumetric wear loss in the FSU group (–36.3 mm^3^) was significantly greater than that in the TC group (–17.6 mm^3^) (*P* < 0.0001), as demonstrated in (Table 3). After the chewing simulation, the worn surfaces showed indentations at the center of the disc where the indenter struck. Images for the samples taken from Geomagic software are shown in (Fig. 3).

**Table 3:** Mean ± standard deviation (SD) values for volumetric measurements (mm^3^) of the TC and FSU groups before and after thermomechanical aging and the mean difference between the two groups

	Before (Mean ± SD) (mm^3^)	After (Mean ± SD) (mm^3^)	Mean Difference (mm^3^)	*P*-value
TC	102.8 ± 5.6	85.1 ± 8.7	-17.6	0.0004*
FSU	90.2 ± 6.6	53.9 ± 4	-36.3	< 0.0001*
*P-*value	< 0.0001*	> 0.0001*	< 0.0001*	

* Indicates a significant difference as revealed by an independent t-test for the volumetric wear test results

### Color change

The results of the color change measured after thermomechanical aging using the CIEDE2000 formula revealed an insignificant difference between the TC ΔE00 values (2.4 ± 0.5) and FSU values (2.1 ± 0.7) (*P* = 0.532), as shown in Table 4. However, both groups were above the clinical acceptability threshold of 1.81 [50].

**Table 4:** Mean ± standard deviation (SD) values for color change (∆E00) of the tested samples in the TC and FSU groups after thermomechanical aging

Group	Color change (∆E00)
TC	2.4 ± 0.5
FSU	2.1 ± 0.7
*P-*value	0.532 (NS)

**NS**; non-significant difference according to the independent t-test

## Discussion

Despite the increase in the variety and frequency of 3D-printed materials for permanent restorations, limited controversial evidence is available regarding their performance and durability [13, 16]. Therefore, the present study aimed to investigate the flexural properties, microhardness, wear resistance, and color stability of 3D-printed nanohybrid resin composites compared with those of milled one, as both are nanohybrid resin composites and have the same claimed clinical applications as stated by the manufacturers. FSU is a commercially available 3D-printed material, and limited research has been conducted to assess its performance.

Characterization of both materials revealed that milled TC contains smaller and regular fillers that are evenly distributed in the organic matrix, whereas FSU contains fewer fillers with large and irregular shapes that are heterogeneously distributed in the organic matrix. Commercially available printable resins contain substantially lower amounts of fillers (3–50%) than resins formulated for subtractive manufacturing (60–86%) [1, 13, 16]. A lower filler load is required for 3D-printing to maintain the liquid consistency needed to provide stable liquid material for printing that does not phase out and avoids sinking of fillers to the bottom of the vat [1, 13]. This facilitates the reproducibility of the prints, where adding a greater amount of filler might increase the viscosity and impair the flow of the resin during the printing process [1, 7].

Evaluating the flexural strength of restorative materials is important for simulating the complex stresses that occur in the oral cavity during service [36]. The results of the present study revealed that, compared with FSU, TC had a significantly greater mean flexural strength, satisfying the requirement of ISO 4049:2019 for flexural strength, which is 100 MPa, but below the manufacturer’s statement of 272 MPa and within the literature statement range (170–254 MPa) [51, 52]. The FSU is below the ISO standard (97.2 MPa) and the manufacturer values (136 MPa). This finding was in agreement with previous studies that reported greater flexural strength in milled than in 3D-printed nanohybrid resin composites used for permanent or temporary restorations [7, 14, 21]. A systematic review reported that the flexural strength of additive resin composites used for definitive restorations ranged from 78 to 125 MPa [13, 16]. However, these results contradict those of other studies that reported that the flexural strength of 3D-printed samples was greater than that of milled samples [17, 28]. The authors attributed the observed results to the printing technique employed, which, in contrast to the milling process, does not induce cracking. Moreover, the curing mechanism and subsequent postcuring of the 3D-printed materials ensure sufficient polymerization [28]. Moreover, other studies reported insignificant differences between 3D-printed and milled resin composites in terms of flexural properties [10, 15].

For better understanding of the stress‒strain behavior of the tested materials, assessment of the flexural modulus has usually been applied. TC had a significantly greater stiffness (7.5 GPa) than FSU did (1.7 GPa), which was in agreement with previous studies [1, 13, 16]. The lower flexural strength and modulus obtained for the 3D-printed resin than for the milled resin in the current study could be explained based on the suggested lower filler volume, as described previously. This reduction in filler content is linearly correlated with the flexural properties of the materials [7, 8]. Additionally, a systematic review reported that the flexure modulus of 3D-printed resin composites ranged from 1 to 7 GPa [13, 16].

High surface hardness is favorable, as it ensures better durability and longevity of the restoration, reducing the risk of surface degradation and failure [36]. The surface hardness results obtained in the present study were in parallel with the flexural property results, where TC (94.6 gf/um^2^) had a significantly greater mean surface hardness value than FSU (31 gf/um^2^). These results were consistent with other studies that reported that compared with their 3D-printed counterparts, milled fixed dental prostheses presented significantly higher surface hardness values [3, 6]. A systematic review reported that the range of microhardness values for 3D-printed resin composites was 14–33 VHN [13, 16]. On the other hand, a study reported higher hardness values for 3D-printed resins than for milled resins and attributed their results to the presence of cross-linked monomers and inorganic fillers in the 3D-printed resins used in their study, which increased their abrasion resistance [29].

Wear resistance is a critical characteristic of dental restorative materials that significantly influences their durability. The wear results revealed that TC exhibited greater wear resistance than FSU did. Wear resistance can be influenced by a multitude of factors, including wear type, load applied, the material and shape of the antagonist, the composition and properties of the material tested, and the application of any aging protocol, such as thermocycling [46]. These factors can lead to wear of the organic matrix and exposure of the inorganic fillers, followed by subsequent filler loss. The tested resin composite materials in this study utilized nano hybrid fillers similar to those found in conventional resin composites. These fillers are both rounded and irregular in shape, with nanosized fillers that can reduce the wear rate relative to larger fillers [48]. These results contradict those of other studies that revealed comparable wear for 3D-printed and milled resin composites [30, 32, 33]. In contrast, a study reported that the 3D-printing technique demonstrated better wear resistance than the milling technique [34]. The variability in laboratory wear testing methods makes it difficult to compare the presented results with those of other studies. The volume of material lost during the interaction between two surfaces is the parameter of choice for assessing the in-vitro wear of resin-based restorative materials. The resin composite specimen shape used in this study presented a limitation, as a flat-disc specimen was used, which lacks the anatomical geometry that simulates the clinical application of these materials. In addition, different manufacturers of resin composites offer a wide range of materials with various chemical formulations that may result in different wear resistances [31, 53].

The mechanical properties results obtained in this study could be further supported by other studies reporting that the 3D-printing process could introduce inconsistencies or defects within the printed layers, which may adversely affect their mechanical properties due to air bubbles entrapment or regions of non-homogeneous microstructure, which may result from inadequate mixing of the resin composite components. In contrast, the milling process of the resin composite block tends to produce a more uniform and dense structure because of its fabrication under controlled and standardized conditions [25, 37–40]. Moreover, the discrepancies observed in the mechanical properties between the current study and previous studies and the manufacturer might be attributed to the variation in the parameters used in the additive 3D-printing technique, including the wavelength and intensity of light curing, orientation of the printing objects, support structure configuration, layer thickness, type of 3D-printer, postprocessing protocol and type of material used for printing [9, 11, 37–41, 49].

Color stability is a crucial factor in ensuring the longevity and esthetic quality of dental restorations. The color change results revealed an insignificant difference in the mean ∆E00 values between the TC and FSU samples after aging. However, both values exceeded the clinically acceptable threshold of 1.8 [50]. These results were further supported by other studies, where the authors recommended the use of 3D-printed and milled resin composites for only 1–2 years as a long-term temporary restoration [26]. However, the lack of statistical significance does not necessarily mean that the two groups are identical or have the same color stability. The insignificant difference between the groups may be attributed to a short period of aging or the use of distilled water only in the thermocycler. Previous investigations reported higher discoloration values after the aging of 3D-printed restorations than after the aging of milled resin restorations [19, 20, 24, 47]. The authors attributed the difference to the chemical composition of the resin used, including the monomer, its polarity, initiator, filler content, crosslinks formed and presence of micropores. Additionally, the presence of hydroxyl side groups can increase the hydrophilicity of resins, which is a critical factor for the water sorption rate and, consequently, color stainability [23].

On the basis of the results obtained in the present study, the null hypothesis was rejected except for color stability, as no significant difference was found between the milled and 3D-printed resin composite materials. A limitation of this study was its in-vitro design, which did not fully replicate the clinical conditions, including pH variations, salivary proteins and enzymes, colorant beverages and mouthwashes. Further research on the printing parameters of commercially available 3D-printed permanent resin composites is recommended. Supplementary investigations regarding the structural and chemical characteristics of 3D-printed materials are still needed. Hence, advanced in-vivo studies are needed to assess the long-term durability of 3D-printed restorations using different materials and printing techniques.

Notably, advancements in materials and techniques are needed to achieve significant improvements in the properties of 3D-printed materials, which could be a possible cost-effective alternative to milling.

## Conclusions

From this study, the following conclusions could be drawn:

1. Milled nanohybrid resin composites have better mechanical performance than 3D-printed nanohybrid resin composites regarding flexural strength, flexural modulus, microhardness and wear.
2. In terms of color stability, both groups were above the acceptability limit of 1.8, suggesting their use only as a long-term temporary restoration in aesthetic areas.

## Electronic supplementary material

Below is the link to the electronic supplementary material.

Supplementary Material 1

## Data Availability

All data generated or analyzed during this study are included in this published article and its supplementary information file.

## Abbreviations

CAD/CAM: Computer Aided Design / Computer Aided Manufacturing
CIEDE2000: International Commission On Illumination Delta E 2000 Color Formula
DLP: Digital Light Processing
EDX: Electron Dispersive X-ray spectrophotometry
FSU: Flexcera Smile Ultra Plus
SEM: Scanning Electron Microscope
SLA: Stereolithography
STL: Standard Triangle Language
TC: Tetric CAD
