# Supplementary material for: Evaluation of a 3D-printed nanohybrid resin composite versus a milled resin composite for flexural strength, wear and color stability
Source: BMC Oral Health. 2025 Apr 15;25:572. doi: 10.1186/s12903-025-05861-2 (PMC11998452; doi:10.1186/s12903-025-05861-2)
Supplement: Supplementary file 1 — Supplementary Material 1 [file 12903_2025_5861_MOESM1_ESM.pdf]

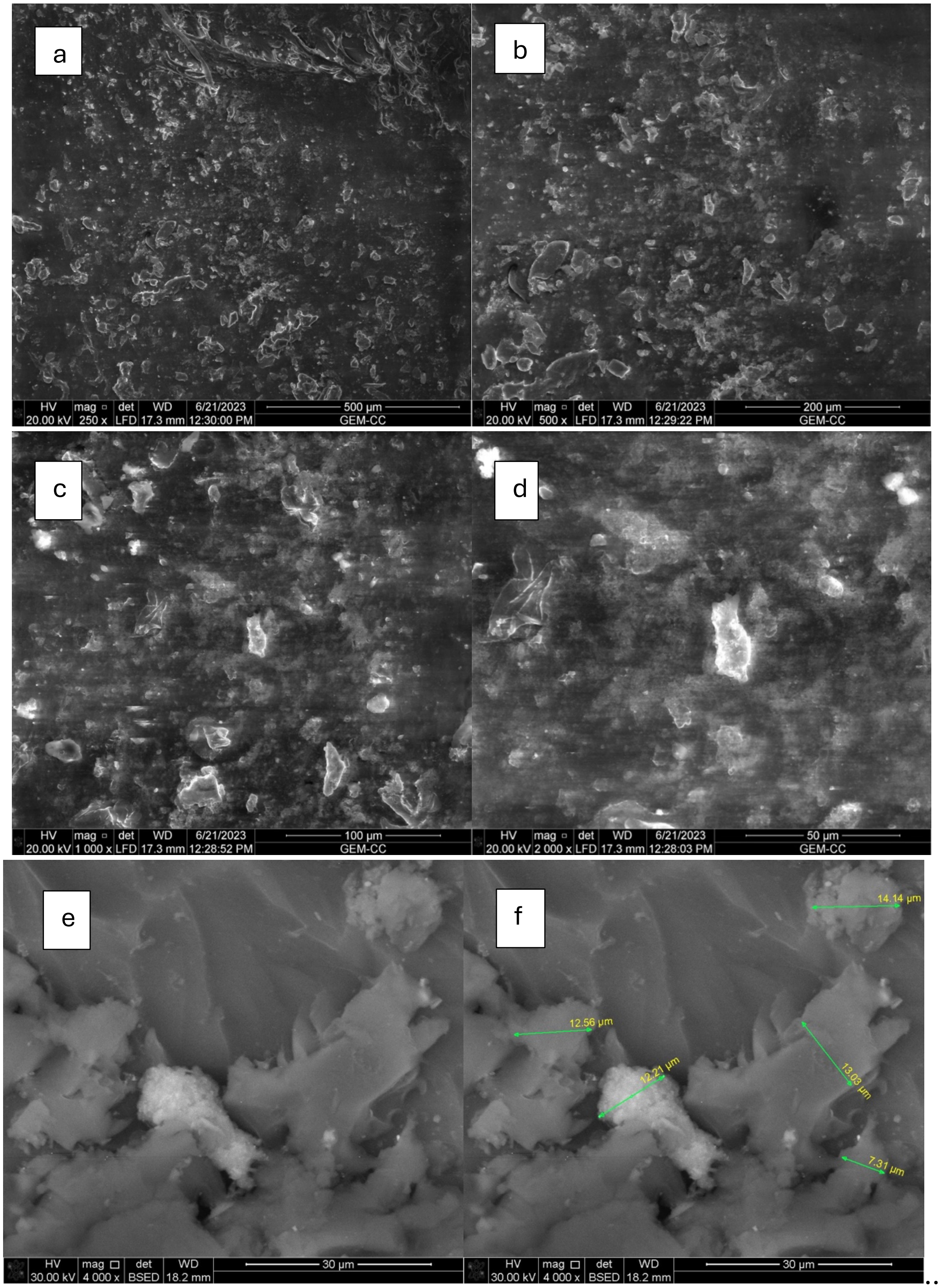

Environmental-SEM images for qualitative analysis of filler sizes and distribution of Flexcera Smile Ultra plus at magnifications of 250x (a), 500x (b), 1000x (c), 2000x (d) and 4000x (e) with large field detector (LFD) and back scattered electron detector (BSED) modes with an accelerating voltage of 20-30 kV. (f) Filler size ranges ~7-14 um. Characterization of this material was conducted at various magnifications using two detectors due to insufficient data available in the literature and from the manufacturer regarding material compositions and structures.

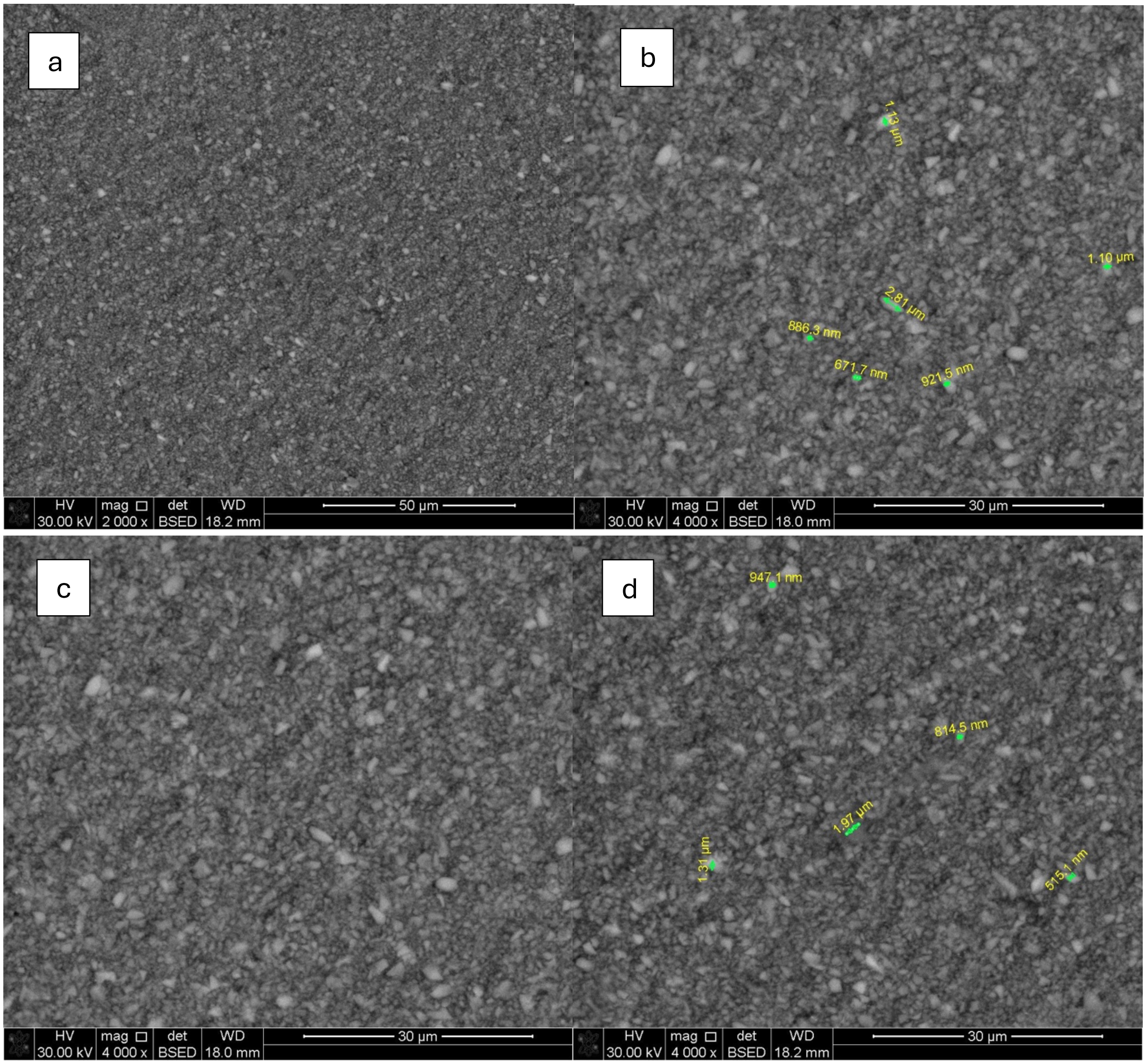

Environmental-SEM images for qualitative analysis of filler sizes and distribution of Tetric CAD at magnifications of 2000x (a) and 4000x (b,c,d) with back scattered electron detector (BSE) mode. (b,d)Filler sizes ranges from <500 nm to > 2um. LFD and different magnifications not used as it gives unclear images for TC. SEM image is closely resembled to that of another study [1] .

EDS analysis for TC

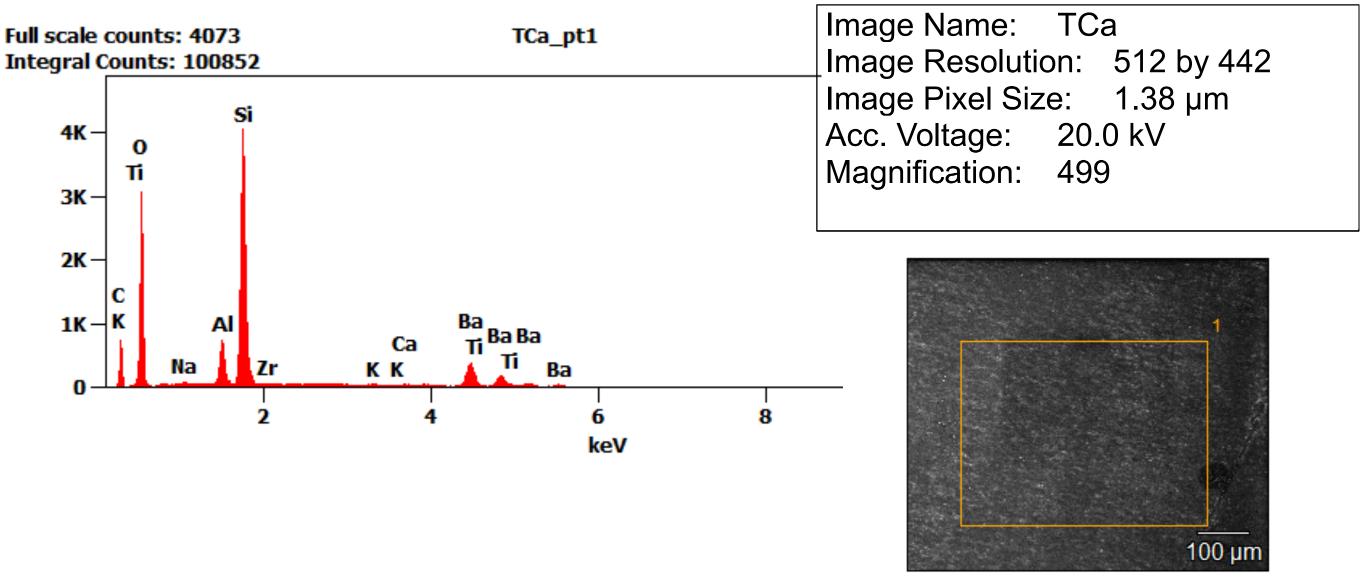

Table shows elemental composition for the Tetric CAD at 500x magnification.

Elements	Intensity	Net Counts	Weight %	Atoms%
C K	12.526	4166	15.704	26.165
O K	44.657	14853	41.429	51.818
Al K	15.15	5039	3.904	2.895
Si K	96.275	32021	23.443	16.703
CaK	0.562	187	0.174	0.087
Ti K	0.652	217	0.285	0.119
Zr L	0.502	167	0.238	0.052
Ba L	22.378	7443	14.825	2.160
			100.000	100.000

The EDS results support what found in other study, which report that the major chemical contents in TC is barium aluminum silicate glass(<1um), silicon dioxide (< 20 nm) as inorganic fillers (71.1 filler wt%) [1]. While the organic matrix represented by carbon and oxygen in Dimethacrylates, Bis-GMA, Bis-EMA,TEGDMA and UDMA[2].

EDS analysis for FSU:

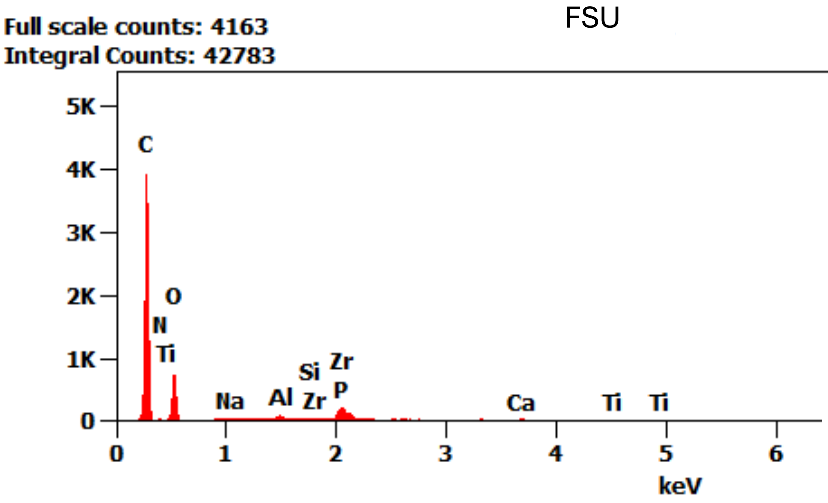

Image Name: FSU
Image Resolution: 512 by 442
Image Pixel Size: 2.76 µm
Acc. Voltage: 20.0 kV
Magnification: 249

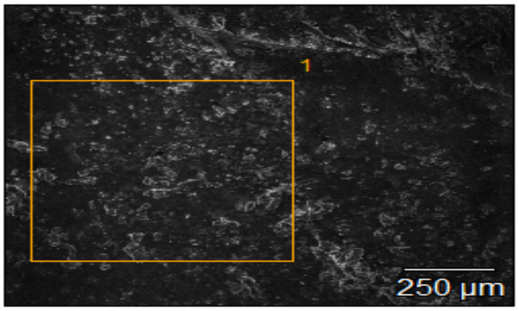

Table show the chemical composition for the FSU taken from the EDS.

Elements	Intensity	Net Counts	Weight %	Atoms%
C K	60.258	18757	40.921	48.881
N K	3.235	1007	16.089	16.480
O K	13.448	4186	36.949	33.133
Na K	0.758	236	0.473	0.295
Al K	1.426	444	0.495	0.263
Si K	0.527	164	0.170	0.087
Ca K	0.761	237	0.352	0.126
Ti K	0.199	62	0.132	0.040
Zr L	8.398	2614	4.419	0.695
			100.000	100.000

EDS results show that the FSU composed mainly from resin matrix (carbon, oxygen and nitrogen) with low filler content (silicon and zirconium) which is needed for 3D printed materials, as increasing filler load will affect the viscosity and the printing process. The manufacturer stated only limited data about the FSU composition (Diphenyl (2,4,6-trimethylbenzoyl) phosphine oxide, methacrylate monomer, methacrylic oligomer & inorganic fillers), and insuficient data availble for the FSU material.
